# A Modified Screening System for Loss-of-Function and Dominant Negative Alleles of Essential MCMV Genes

**DOI:** 10.1371/journal.pone.0094918

**Published:** 2014-04-14

**Authors:** Madlen Pogoda, Jens B. Bosse, Karl-Klaus Conzelmann, Ulrich H. Koszinowski, Zsolt Ruzsics

**Affiliations:** 1 Max von Pettenkofer-Institut, Ludwig-Maximilians-Universität, Munich, Germany; 2 DZIF - German Center for Infection Research, Munich, Germany; Queen's University, Canada

## Abstract

Inactivation of gene products by dominant negative mutants is a valuable tool to assign functions to yet uncharacterized proteins, to map protein-protein interactions or to dissect physiological pathways. Detailed functional and structural knowledge about the target protein would allow the construction of inhibitory mutants by targeted mutagenesis. Yet, such data are limited for the majority of viral proteins, so that the target gene needs to be subjected to random mutagenesis to identify suitable mutants. However, for cytomegaloviruses this requires a two-step screening approach, which is time-consuming and labor-intensive. Here, we report the establishment of a high-throughput suitable screening system for the identification of inhibitory alleles of essential genes of the murine cytomegalovirus (MCMV). In this screen, the site-specific recombination of a specifically modified MCMV genome was transferred from the bacterial background to permissive host cells, thereby combining the genetic engineering and the rescue test in one step. Using a reference set of characterized pM53 mutants it was shown that the novel system is applicable to identify non-complementing as well as inhibitory mutants in a high-throughput suitable setup. The new cis-complementation assay was also applied to a basic genetic characterization of pM99, which was identified as essential for MCMV growth. We believe that the here described novel genetic screening approach can be adapted for the genetic characterization of essential genes of any large DNA viruses.

## Introduction

Traditionally, irradiation or chemicals were used to increase the mutation rate during replication, and interesting phenotypes were subsequently investigated regarding the causative genetic change [1]. However, comprehensive genetic analysis of herpesviruses by this approach was not feasible and the application of traditional methods of molecular cloning has long been limited due to the size of their genomes. Primarily the establishment of genetic systems that permit directed mutagenesis of the gene of interest in its genomic context facilitated the generation of virus mutants in order to analyze resulting phenotypic alterations (reviewed in [2]).

Functional analyses of isolated genes were enabled by cloning of viral fragments that could be modified *in vitro* and re-introduced into the viral genome. Yet, the recombination frequency is low exploiting the cellular recombination machinery and the majority of produced viruses do not carry the desired mutation (reviewed in [3]). The disadvantages of uncontrollable cellular recombination was overcome, when full length herpesvirus genomes were cloned as infectious bacterial artificial chromosomes (BAC), which was pioneered for the murine cytomegalovirus (MCMV) [4], and subsequently adapted to numerous herpesviruses such as HSV-1, PrV, HCMV, EBV and MHV68 [5]–[9], and also to adenoviruses [10]. Using BACs, viral genomes can be readily modified using techniques developed for bacterial genetics. Besides, the expression of viral functions is not required for the maintenance in *E.coli*, decreasing the risk of unwanted changes due to frequent sub-culturing of viral progeny. The modified DNA is transfected subsequently into permissive eukaryotic cells to reconstitute infectious virus, which results in pure, clonal viral populations, and even allows the production of attenuated mutants. While the function of non-essential genes can be studied in the virus context using deletion and loss-of-function mutants, this approach is not applicable directly for essential genes. The study of null- or non-functional mutants of essential genes requires functional analysis by *trans*-complementation or the application of dominant negative (DN) mutants (reviewed in [11], [12]).

Viruses with inactivating mutations in essential genes can be propagated only if the target gene is provided in addition. This can be realized by expressing the complementing feature separately from the virus genome (*trans*-complementation), for example by a modified helper cell line or helper viruses. *Trans*-complementation is commonly used for early gene products, which are required in small amounts for viral replication and whose expression is not detrimental for the cell. Alternatively, the complementing element can be inserted into the viral genome at an ectopic position (*cis*-complementation), often accompanied with regulating features [3], [13].

Essential genes are not studied easily using traditional approaches because mutant viruses are difficult to reconstitute. However, functional inactivation of such proteins by co-expression of DN mutants makes them amenable to comprehensive genetic analysis [12], [14]. DN alleles are able to induce the null phenotype in the presence of the wt gene product and thus permit functional analysis of essential genes. A DN mutant can induce the same phenotype as the deletion of that gene. However, investigation of deletion mutants of essential genes requires propagation on a *trans*-complementing cell line. This is not necessary for DN mutants, which can be analyzed by conditional expression in the virus context. Moreover, null-mutants only reveal the dominant role of a protein. DN alleles, in contrast, have the potential to arrest viral pathways at different stages, thereby addressing multiple essential functions of a protein [12].

Thus, isolation and characterization of DN alleles became a standard procedure in genetics. Using in-depth functional knowledge and detailed experimental information regarding protein structure, such inhibitory mutants can be created by targeted introduction of crucial, but subtle mutations or by deleting a domain that represents an independent folding entity [12], [14], [15]. However, so far such data are limited for the majority of herpesvirus proteins, impeding a knowledge-based construction of DN mutants. Consequently, the entire coding sequence of a target gene needs to be subjected to random mutagenesis to identify potential DN candidates [16]–[19]. A viral conditional expression system has been adapted to MCMV, in which the two elements required for conditional gene expression – regulated transcription unit as well as regulator – were integrated as one cassette into the viral genome [20], [21]. This *tet*-regulated system has successfully been applied to the conditional expression of both essential genes and DN alleles in the virus context [15]–[17], [19].

For comprehensive mutagenesis of a single viral gene, the open reading frame (ORF) is subcloned and mutagenesis performed on plasmid basis. The modified plasmids are inserted individually into a BAC deficient for this gene and the targeting of essential and non-essential sites identified by its ability to restore viral reconstitution. Loss-of-function mutants are then tested in a similar screen for their potential to inhibit the wt protein function by using a wt BAC as acceptor [16], [19]. Although DN mutants are potent tools in genetic analyses, their identification using this strategy of two genetic screens, which are both based on individual flip-in in *E.coli* followed by BAC selection and virus reconstitution, is time-consuming and labor-intensive. Thus, new approaches are called for that decrease the screening effort.

In this report, we describe a fast and efficient system for the identification of inhibitory alleles of essential MCMV genes that is based on Flp-mediated recombination in permissive mammalian cells instead of *E.coli*. As for the previous two-step screening, first an MCMV BAC lacking an essential gene served as acceptor genome, which allows the identification of complementing and non-complementing mutants, provided in the context of a donor plasmid for Flp-mediated recombination. The non-functional mutants were then tested for their inhibitory capacity in a next round using an acceptor MCMV genome, which is wt at the locus of interest. We tested the new screening approach with a reference set of M53 mutants and, finally, we demonstrated the applicability of this system by testing a set of M99 mutants. Homologs of pM99 can be found in all herpesviruses and have been shown to be crucial in secondary capsid envelopment [22]–[25].

## Materials and Methods

### Cells and Viruses

Murine embryonic fibroblasts (MEF) were propagated according to the standard protocol as described [26], [27], NIH/3T3 murine fibroblasts (ATCC CRL-1658), and 293 cells (ATCC CRL-1573) were cultured as described previously [28], [29]. Flpe-expressing cells (Flpe-NIH) were generated by stably transfecting commercially available NIH/3T3 (ATCC CRL-1658) cells with the pCAGGS-Flpe plasmid (Gene Bridges GmbH, Heidelberg) [30] and cultured in the presence of 3 µg/ml puromycin in Dulbecco's modified Eagle's medium (DMEM) supplemented with 10% fetal calf serum (FCS), 0.3% L-glutamine, and 0.05 mM nonessential amino acids (Invitrogen). All MCMV mutants were derived from the parental MCMV bacterial artificial chromosome (BAC) pSM3fr-Δ1-16-FRT, in which the dispensable genes *m01* to *m16* are deleted and a FLP recombination target (FRT) site is inserted. This BAC gives rise to the virus MCMV-Δ1-16-FRT after transfection of permissive cells, which replicates in tissue culture with wt characteristics [19]. MCMV BACs were reconstituted to viruses by transfecting MEF with 1.5 µg purified BAC DNA using SuperFect Transfection Reagent (Qiagen) according to the manufacturer's instructions, and supernatants were harvested when the cells were completely lysed. The infectivity of the virus inocula was quantified by a standard plaque assay on MEF [31].

### Characterization of Flpe expression and activity

To confirm Flpe expression, approximately 1×10^6^ Flpe-NIH were harvested and total cellular RNA was purified using the RNeasy Mini Kit (Qiagen). Then, 500 ng of the purified RNA was reverse transcribed using the Superscript RNase H – Reverse Transcriptase (Invitrogen) according to the manufacturer's instructions using Oligo (dT)_20_ Primer (Invitrogen). The resulting cDNA was PCR-probed using a primer pair specific for the *flpe* gene (Flpe-for/Flpe-rev; for primer sequences refer to Table S1).

To confirm the functional activity of the Flpe recombinase with a plasmid-based recombination assay, NIH/3T3 and Flpe-NIH cells were nucleofected with 500 ng of the plasmid pCP15 [32]. This plasmid contains, in addition to an intact beta-lactamase ORF, a gene conferring resistance to kanamycin (kanR) flanked by two unidirectional FRT sites, which is deleted by active Flpe recombinase. Cells were harvested at 1 dpt, total DNA was isolated using the DNeasy Blood and Tissue Kit (Qiagen) and PCR-probed for Flpe-mediated recombination of pCP15. Three PCRs were designed to check Flpe activity. In the R reaction the primer REC15for anneals upstream of the first FRT site, the reverse primer REC15rev downstream of the second FRT site. The resulting product in the non-recombined plasmid has a length of approximately 1.8 kbp. In contrast, if the kanR cassette was removed by Flpe-mediated recombination, the resulting amplicon of the R reaction has a size of 434 bp. An alternative reverse primer, NR15rev, attaching right behind the first FRT site, was used together with REC15for of the R reaction to detect non-recombined DNA specifically (N reaction). This 358 bp amplicon in the N reaction spans the upstream FRT site as well as the 5‘ region of the kanR cassette. The plasmid load was controlled by the B reaction which amplifies a 340 bp fragment of the beta-lactamase gene (primers BLAfor and BLArev). All PCRs were set up with approximately 400 ng of total DNA as template.

### Plasmids

The acceptor plasmid pDEST-pac was generated by inserting the amplicon PCR-MCMVpac (PCR on pSM3fr-Δ1-16-FRT using primers MCMVpac_for/MCMVpac_rev) into pEF5/FRT-V5-DEST (Invitrogen) after treatment with *Nde*I and *Rsr*II. To generate rescue plasmids for the CIA, the ORFs encoding wt M53 and the M53 mutants i115, i128, i146, i207, i220, i313, and s309 were excised from the pO6-ie-derived vectors [33] and inserted into pENTR11 (Invitrogen) using *Kpn*I/*Not*I. Subsequently, the genes were transferred from the pENTR11 vector into pDEST-pac employing the LR reaction of the Gateway system (Invitrogen) according to the manufacturer's instructions.

Rescue plasmids constitutively expressing pM99 were constructed as follows: The plasmid encoding Flag-tagged pM99 expressed under control of P_CMV_ and IRES-coupled GFP expression was produced by ligating the large fragment of pDNS-M99F-iChe and the small fragment of pIRES2-AcGFP1 (Clontech Laboratories, Inc.) after treatment with *Not*I and *Sal*I, giving rise to pDNS-M99F-igfp. pDNS-M99F-iChe was cloned by inserting the small fragment of pMA-T-M99F (synthesized by GeneArt, Invitrogen) into pO6-A5-DNS-Che after cleavage with *Nhe*I and *Sal*I. The pO6-A5-DNS-Che expression vector, in turn, was assembled from the large fragment of pIRES-Che-pA (kind gift of Sigrid Seelmeir) and the small fragment of pDEST-pac following *Nde*I/*Rsr*II treatment. The resulting plasmid was cut with *Pac*I, blunted using T4 DNA polymerase, re-ligated, cleaved with *Afe*I and *Mlu*I, and ligated to the large fragment of pO6-A5-CMVgfp (kind gift of Simone Boos) treated with the same enzymes. pDNS-M99-igfp was generated by inserting the *Nhe*I/*Sal*I-treated amplicon PCR-M99 (PCR on pDNS-M99F using the primer pair M99syn-for/M99rev) and the small fragment of *Not*I/*Sal*I-cleaved pIRES2-AcGFP1 into *Nhe*I/*Not*I-opened pDNS-M99F. Expression plasmids for pM99 regulated by P_M99_ were generated by ligating the amplicon PCR-P(M99) encoding the M99 promoter region (PCR on pSM3fr-Δ1-16-FRT using primers P(M99)-for/P(M99)-rev) and pDNS-M99F-ifgp after treatment with *Nhe*I and *Pac*I, giving rise to pDNS-PM99F-igfp. The version lacking the Flag-tag was assembled by inserting the *Nhe*I/*Sal*I-treated amplicon PCR-M99 (PCR on pDNS-M99F using primers M99syn-for/M99-rev) and the *Nhe*I/*Pac*I-treated amplicon PCR-P(M99) into *Pac*I/*Sal*I-opened pDNS-M99F-igfp. The plasmid encoding pM99 lacking the N-terminal glycine (ΔGly2) was generated by inserting the PCR-amplified (PCR on pDNS-PM99-igfp using primers M99rev/M99ΔGly2for) into the large fragment of pDNS-PM99-igfp after treatment with *Nhe*I/*Sal*I. The rescue plasmid expressing pM99 lacking the potential pM94 binding site (Δ94) was ligated from the large fragment of pDNS-PM99-igfp, the *Pac*I-treated PCR product (primers P(M99)-for and M99ΔM94-5′rev) and the *Sal*I-cleaved amplicon (primers M99ΔM94-3′for and M99rev). The pM99 mutant lacking the acidic cluster (ΔAC) was generated similarly, but using the primer pairs P(M99)-for/M99ΔAC-5′rev and M99ΔAC-3′for/M99rev. PCR template was pDNS-PM99-igfp for the latter three plasmids.

### Construction of recombinant viral BACs

Recombinant BACs were based on pSM3fr-Δ1-16-FRT [19]. The BACs lacking the ORFs for M56 (ΔM56), M99 (ΔM99), M104 (ΔM104), and the packaging signals (Δpac) were generated by homologous recombination using PCR products as described previously [34]. The M99 ORF was also deleted from pSM3fr-Δ1-16-FRT-SCPiChe, which was generated on the basis of pSM3fr-Δ1-16-FRT by insertion of an mCherry ORF coupled with an IRES to the 3' untranslated region of the ORF encoding the smallest capsid protein (SCP, M48.5), giving rise to the acceptor BAC pSM3fr-Δ1-16-FRT-SCPiChe-ΔM99 (SCPiChe-ΔM99). Construction of *cis*-complemented genomes in *E.coli* by Flp-mediated recombination was performed as described previously [13].

### Nucleofection

To perform nucleofection reactions, 0.5 µg BAC DNA was mixed with 0.5 µg plasmid DNA. Low-passage Flpe-NIH were harvested, pelleted at 90×*g* for 10 min and suspended in aliquots of 5×10^5^ cells in 20 µL solution SG of the Lonza SG Cell Line 96-well Nucleofector Kit (V4SC-3096) onto the DNA mixtures. Samples were mixed and transferred air bubble-free into the Lonza electroporation stripes and nucleofected by means of the Amaxa 96-well Nucleofection System using the program EN-158. After 10 min recovery at room temperature in the electroporation cuvettes, cells were mixed with 80 µL supplemented medium and aliquots of 1×10^5^ Flpe-NIH were seeded onto 12- or 24-well plates, which already contained 3×10^5^ non-modified NIH/3T3. Plates were incubated at 37°C for 6 days, before cells were fixed and processed for microscopic analysis. For the experiments which were performed to measure the plaque sizes we used tha specially selected FCS batch (from PAA) to support slow growth of NIH3T3 cell.

### Immunofluorescence microscopy

Nucleofected cells grown on multi-well plates were fixed after 6 days of incubation with 4% paraformaldehyde (PFA) for 15 min at 37°C, permeabilized by treatment with 0.1% Triton X–100 for 15 min, blocked for 60 min with 3% bovine serum albumin (BSA), and stained with antibodies specific for IE1 (pp89) or the major capsid protein (MCP). These were in turn reacted with the appropriate Alexa Fluor-coupled secondary antibodies (Molecular Probes). Photographs were taken on a Zeiss Axiovert 25 with 488-, 543-, and 633-nm laser.

## Results

### Strategy for a direct cis-complementation assay

To enable a direct screening in cells which allows us to avoid the construction of each individual mutant in *E.coli*, the original system [16], [19] had to be modified at several stages. In both strategies the acceptor genome is a viral BAC that lacks the gene of interest and carries an FRT site for recombination. Also, the same donor plasmid carrying a wt or mutant ORF of the gene of interest and a second FRT site, which has been adapted for several studies [15], [16], [17], [19], can be used here. This donor plasmid is termed now rescue plasmid to emphasize its function, namely to provide a potential complementing feature. In the original approach these two genetic elements were recombined in *E.coli* [13]. Here, the two constructs are introduced into permissive cells that express Flp recombinase to facilitate the unification of the two above described genetic elements. Since Flp-mediated recombination is reversible, insertion of the rescue plasmid into as well as excision from the acceptor BAC will happen in those cells. Moreover, under non-selective conditions, the excision reaction is kinetically favored over the integration [35]. In order to prevent an overwhelming excision activity during the assay the Flp-expressing cells were mixed following transfection with normal permissive cells at a ratio of 1∶3. These normal cells will become infected with the viruses reconstituted in the Flpe-NIH and amplify the complemented genomes in order to allow plaque formation in the absence of Flp recombination. In a pool of pure Flp-expressing cells, any free viral genome would constantly be prone to recombination events, i.e. flip-in and flip-out. By mixing the Flp-expressing cells, the recombined viruses can infect the normal cells and the viral DNA is not targeted by the recombinase during amplification.

**Figure 1:**
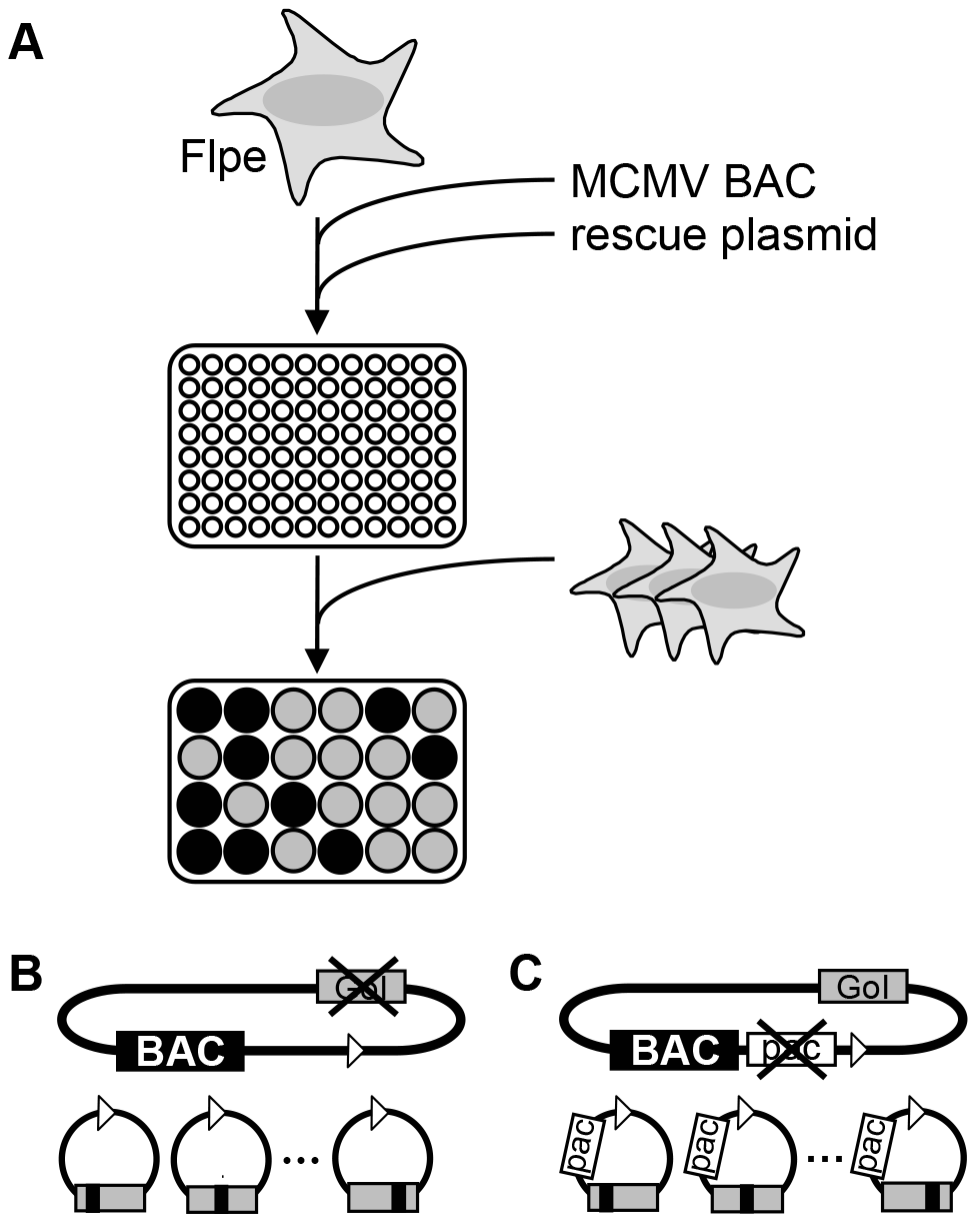
Principle of the cell-based screening system. **(A)** Murine fibroblasts stably expressing enhanced eukaryotic Flp recombinase (Flpe) were mixed with BAC DNA, which lacks an essential feature and carries an FRT site for homologous recombination, and a shuttle plasmid, which carries a second FRT site, and transfected by nucleofection. Transfected cells were mixed with non-modified fibroblasts at a ratio of 1∶3, seeded on multi-well plates and viral plaque formation was monitored. The unification of the shuttle plasmid and the target genome takes place within the host cell in the presence of Flpe expression. **(B)** Cell-based complementation assay (CCA). The MCMV target BAC is deficient for an essential viral gene (GoI, gray box), which is mutated and subcloned into the rescue plasmid (gray box with black line). Viral reconstitution (formation of plaques) was expected if the mutated gene was able to complement the wt protein function (depicted as gray wells in (A)), whereas the absence of plaque formation using the same conditions would indicate non-complementing mutants (black wells in (A)). **(C)** Cell-based inhibitory assay (CIA). The target BAC carries a wt copy of the GoI (gray box), but the essential packaging sequences (pac; white box) were removed and cloned into a rescue plasmid, which also encodes mutants of the GoI (gray box with black line). Reduced viral reconstitution was expected when the mutated form of the GoI was inhibitory for the wt protein (depicted as black wells in (A)), whereas non-inhibitory mutants would allow wt-like reconstitution (gray wells).

The transfection needs to be carried out efficiently, since it targets a relatively small number of cells to allow multiplication. We therefore decided to use nucleofection in a multi-well plate format. Nucleofection is a non-viral transfection technology, which permits efficient delivery of transfected DNA into the nucleus, thus providing high nuclear plasmid concentrations [36]–[38]. The nucleofected Flp-expressing cells, mixed with non-transfected NIH/3T3, are plated on multi-well plates and viral plaque formation is observed. If the rescue plasmid carried a functional, complementing version of the deleted gene, virus should be reconstituted successfully after the fusion of the acceptor BAC and the rescue plasmid, which will be detected by plaque formation. In contrast, if the rescue plasmid carried a non-functional version of the deleted gene, virus will not be reconstituted and viral plaques will not appear. A schematic representation of this screen is depicted in Figure 1.

### Construction of a Flp-expressing stable cell line for MCMV *cis*-complementation

**Figure 2:**
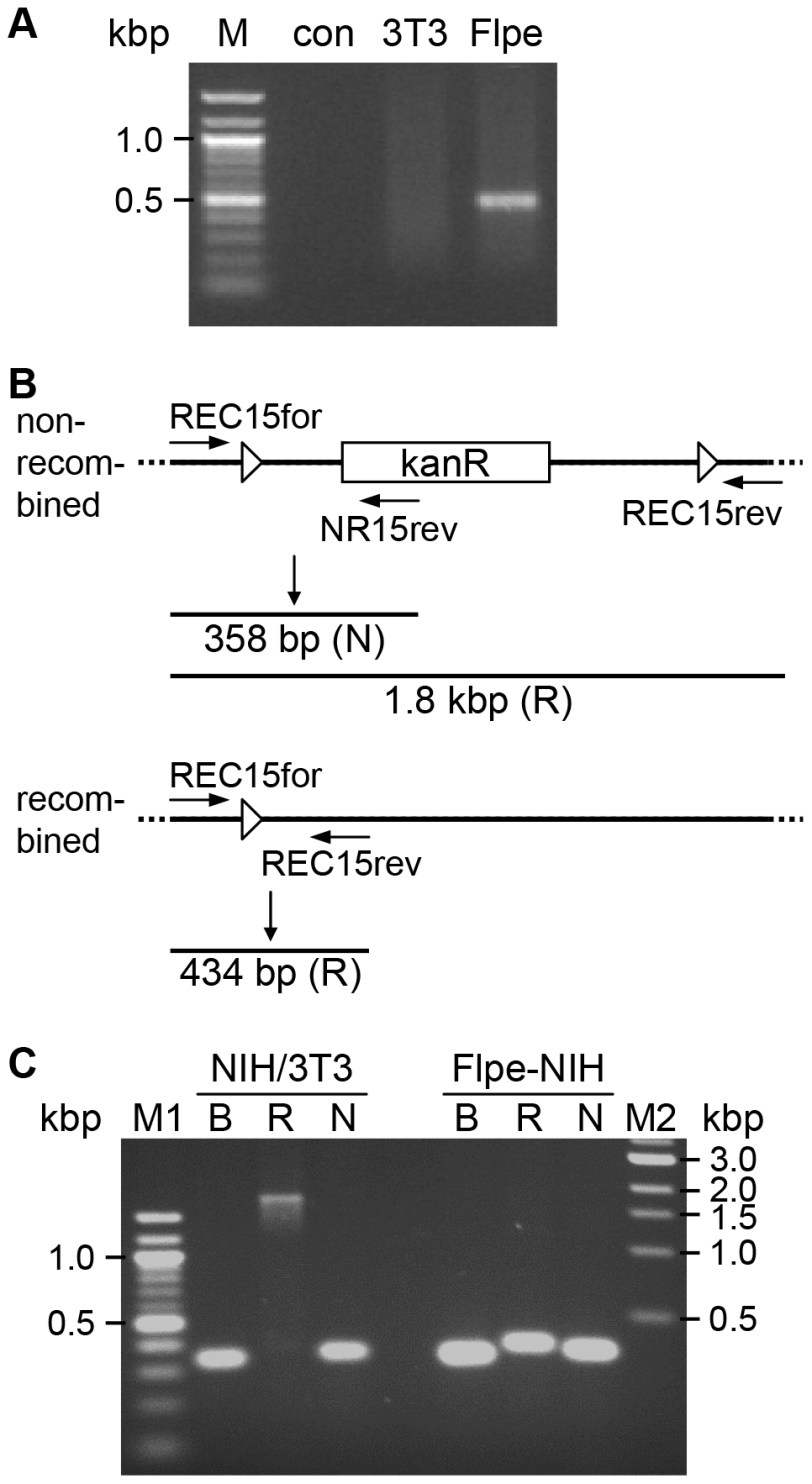
Flpe recombinase is expressed and functional. **(A)** Total RNA was isolated from NIH/3T3 (3T3) and Flpe-NIH cells (Flpe), reverse transcribed and PCRed using primers specific for the *flpe* gene. The control reaction (con) did not contain any template. M, 100 bp DNA ladder. **(B)** Schematic representation of the N and R reaction. For non-recombined pCP15, the R reaction results in a 1.8 kbp fragment, whereas the N reaction amplifies a 358 bp fragment. In recombined plasmids, the R reaction produces a 434 bp amplicon. **(C)** NIH/3T3 and Flpe-NIH cells were nucleofected with the plasmid pCP15. Cells were harvested at 1 dpt, total DNA was isolated and PCR-probed for Flpe-mediated recombination of pCP15. The recombination is probed by the R and the N reactions (R, N) as described above. The plasmid load was tested by the B reaction (B). M1, 100 bp DNA ladder; M2, 1 kbp DNA ladder.

To provide Flp for the site-specific recombination in the first step of the cell-based *cis*-complementation assay, we generated a cell line on the basis of NIH/3T3 murine fibroblasts by stable transfection of the expression plasmid for enhanced Flp recombinase (Flpe), which is a modified form of the original Flp evolved by cycling mutagenesis and catalyzes the recombination reaction more efficiently at 37°C [30]. Single cell clones were picked, Flpe mRNA expression was tested by RT-PCR, and a positive cell clone was grown up to a cell line named Flpe-NIH. Flpe expression was also continuous during the establishment of the cell line and confirmed in parallel to each experiment. A representative observation is shown in Figure 2A.

We also tested the functionality of the expressed recombinase by a plasmid-based recombination assay. For this, NIH/3T3 and Flpe-NIH cells were nucleofected with the plasmid pCP15. This plasmid contains a gene conferring resistance to kanamycin (kanR) flanked by two unidirectional FRT sites, which is deleted by active Flpe recombinase [32]. To check Flpe activity, we used three different PCRs (Fig. 2B). In the R reaction the entire recombination cassette is amplified, which results for the non-recombined plasmid in a product of approximately 1.8 kbp. In contrast, if the kanR cassette was removed by Flpe recombination, the resulting amplicon has a size of 434 bp. This short amplicon competes out the larger products. Therefore, to characterize the recombination activity further, an alternative reverse primer, attaching right downstream of the first FRT site, was used together with the forward primer of the R reaction. This 358 bp amplicon spans the upstream FRT site as well as the 5‘ region of the kanR cassette and should only be absent if all plasmids are recombined (N reaction).

Nucleofected cells were harvested at 1 dpt, total DNA was isolated and PCR-probed for Flpe-mediated recombination of pCP15 (Fig. 2C). No plasmid recombination was observed in the absence of Flpe in NIH/3T3 cells by the R reaction, as demonstrated by the presence of the 1.8 kbp fragment. In contrast, a substantial number of the plasmids were recombined at the point of DNA extraction in Flpe-NIH cells, as detected by the presence of the shorter amplicon in the R reaction. In addition, in both conditions the N reaction was positive. The same result was observed up to 5 dpt (data not shown), indicating that a complete flip-out would not occur in these cells. Comparable plasmid load was detected by the presence of the control amplicon in the B reaction in both samples (Fig. 2C).

### *Cis*-complementation of essential MCMV genes in cells

**Figure 3:**
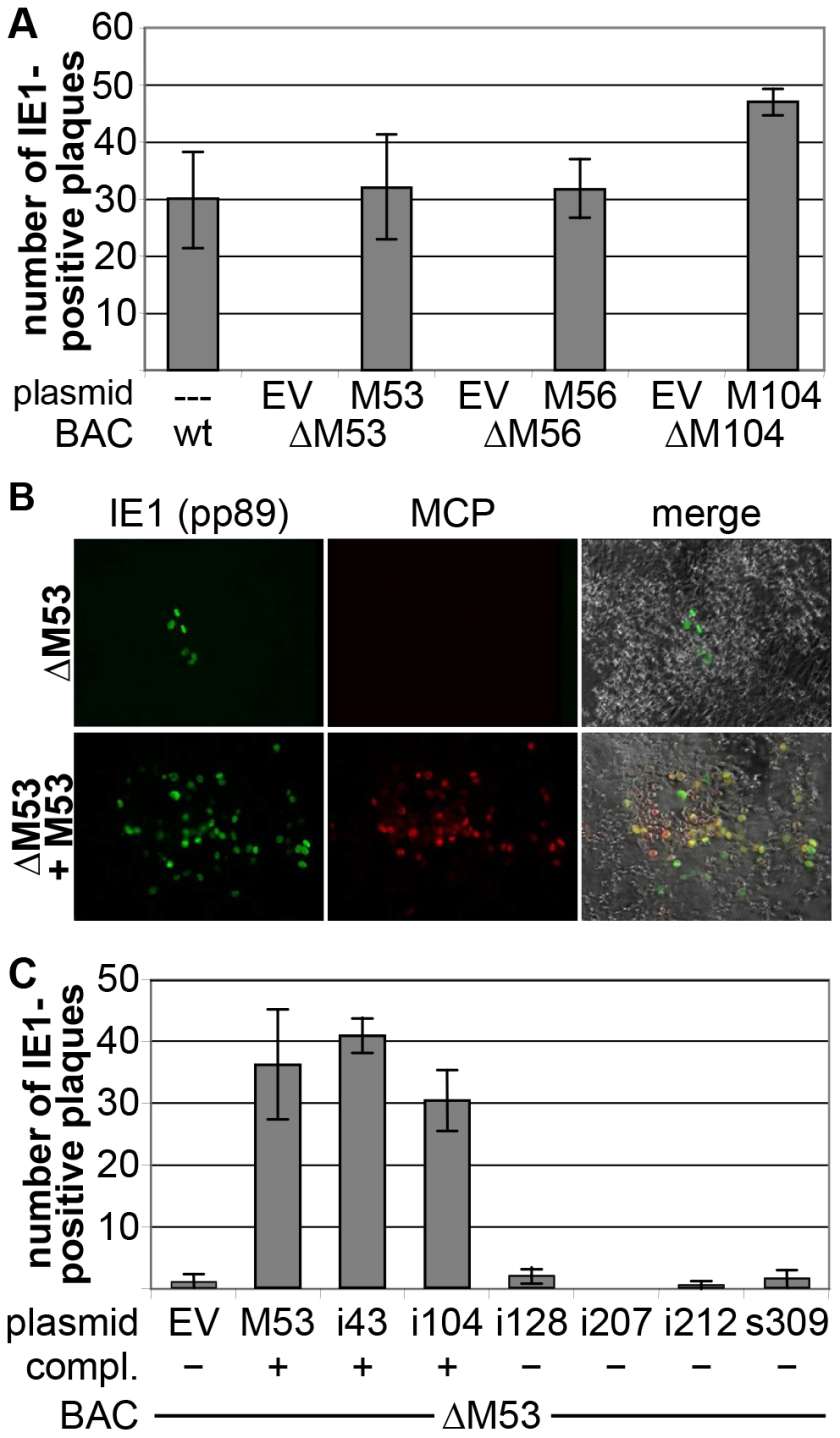
Verification of the cell-based complementation assay. **(A)** Flpe-NIH were nucleofected with the indicated BACs and plasmids and mixed with NIH/3T3. Samples were fixed at 6 dpt, stained with an antibody specific for the IE1 protein and IE1-positive plaques quantified. Depicted are average and SD of triplicate samples of two independent experiments. EV, empty vector. **(B)** Flpe-NIH were treated as in (A), stained with antibodies specific for the IE1 (pp89) and the major capsid protein (MCP) and images taken using a fluorescence microscope. **(C)** Flpe-NIH were nucleofected with the ΔM53 BAC and a rescue plasmid expressing the indicated M53 mutants and treated as in (A). Depicted are mean and SD of duplicates of two experiments. Mutants were generated by transposon-based random mutagenesis and described in [33]. Their capacity to complement the loss of the wt pM53 protein is indicated below the diagram. EV, empty vector; i, insertion of five amino acids; s, stop mutation at this position.

In order to test the basic principle, Flpe-NIH were nucleofected with MCMV BACs which were generated by deletion of the essential genes M53, M56, and M104 from pSM3fr-Δ1-16-FRT [19], and the respective FRT site-containing rescue plasmids constitutively expressing the complementing gene. As control all acceptor constructs were co-transfected with the empty recue vector (Fig. 3A). The M53 gene is known to be essential and was subjected to all ectopic complementation-based screens published before [17], [33], providing a possibility to compare the fidelity of the new assay to the old standards. The essentiality of pM56, the MCMV homologue of the pUL28 herpesvirus protein family encoding the large subunit of the viral terminase [39], was confirmed by transfecting MEF with an MCMV BAC lacking the M56 ORF (ΔM56). No viral plaques were observed for six weeks (data not shown).

The nucleofected cell were seeded onto 12-well plates, 6 dpt the samples were stained with an antibody specific for the IE1 protein pp89 and the number of fluorescent foci which were larger than 15 cells was quantified (Fig. 3A). No IE1-positive plaques were detected after nucleofection with the three deletion BACs, confirming the essentiality of those genes for viral reconstitution. Occasionally, small, few cells containing foci of IE1-positive cells were observed, which did not show any cytopathic effect (CPE) characteristic for lytic MCMV infection (an example is shown in Fig. 3B). Conversely, co-transfection of each deletion BAC with its respective rescue plasmid resulted in robust plaque formation at 6 dpt. By the IE1-staining, large positive foci were observed that were either surrounding cell free areas (plaques) or showed characteristic CPE in the center indicating lytic MCMV infection. Whereas for the complementation of the M53 and the M56 deletion an average of 32 fluorescent foci was detected, complementation of the M104 deletion resulted in an average of 47 plaques (Fig. 3A). In addition to the staining detecting pp89, a sample of M53 complementation was probed with an antibody specific for the major capsid protein (MCP), a gene product expressed late in the replication cycle [20]. In the few pp89 positive small foci found in the control transfection no MCP-specific signal was observed, verifying that in this foci virus reconstitution did not take place. In contrast, the cells surrounding the viral plaques in the sample co-transfected with the M53-expressing rescue plasmid displayed strong signals for both pp89 and MCP, confirming the productive virus cycle (Fig. 3B).

These data showed that complementation reproducibly and specifically takes place in our new set-up, therefore we coined the system cell-based *cis*-complementation assay (CCA).

### Identification of non-complementing mutants using the cell-based *cis*-complementation assay (CCA)

The previous experiments indicated that the complementing gene can rescue the null mutants in the new nucleofection-based assay. The next step was to investigate whether this system could be used to test the functionality of mutant alleles. For this, a number of mutants of an M53 library generated by transposon mutagenesis was selected and tested as described above (Fig. 3C). The M53 mutant library has been studied extensively and the capacity of each mutant to complement the M53 deletion has been tested with the previous cis-complementation screens [17], [33].

As before, few small IE1-positive foci were detected upon co-transfection of the ΔM53 BAC with the empty rescue plasmid, but viral reconstitution was not observed, whereas complementation of the M53 deletion with the wt M53-expressing rescue plasmid resulted in robust plaque formation. Expression of the complementation-competent mutants i43 and i104 also led to the formation of viral progeny, ranging around wt level. The mutants i128, i207, i212 and s309 failed to complement the M53 deletion in the previous screen [33]. This observation was confirmed using the new assay, where no viral reconstitution was detected for any of these mutants (Fig. 3C).

Using the M53 mutant library as reference set, the results observed in this screen correlated exactly with the observations published before [17], [33]. Thus, the CCA is applicable for the identification of non-complementing mutations, and can be used to screen for non-functional mutants of essential genes.

### Identification of inhibitory mutants

The basic CCA screen aimed at identifying mutants that fail to restore viral reconstitution in the absence of the gene of interest. Only such non-complementing mutants have the potential to inhibit the wt protein functionally by a dominant negative mechanism [12]. However, most of the non-functional mutants lack the potential to inhibit the wt function [16], [17], [19]. To identify inhibitory mutants, the non-functional mutants from the basic CCA have to be re-analyzed in a second assay, previously coined the inhibitory screen [16].

Based on the principle of the CCA a novel cell-based inhibitory assay (CIA) was developed. However, for a successful inhibitory assay the mutants need to be inserted into all reconstituted viral genomes. Otherwise, if the co-transfection with the rescue plasmid is not 100%, the escape of viral progenies derived from a non-recombined wt-like BAC is possible. To ensure that only recombined BACs are reconstituted to virus, an additional essential genetic element which cannot be complemented *in trans* was transferred from the acceptor BAC to the rescue plasmid. Such an essential *cis* element is provided by the packaging signals (*pac*). Each MCMV genome is terminally flanked by two *pac* sequences. These are recognized by the viral terminase during the encapsidation process, which induces cleavage of the concatemeric viral DNA, resulting in a unit length genome that is finally packaged [40]. To ensure proper cleavage, the *pac* sequences have to be present in the genome, i.e. only genomes with the inserted element will be packaged and reconstituted to virus. To this end, the terminal region spanning the two *pac* signals (nt 230,000 to 230,100 fused to nt 1 to 100) was removed from the MCMV BAC, resulting in the new acceptor BAC Δ*pac*, and cloned into the rescue plasmid, giving rise to pDEST-pac. The rescue plasmid carried a transcription unit for the constitutive expression of the gene of interest under control of the eukaryotic elongation factor 1α (EF-1α) promoter, which has been shown to be superior to the HCMV IE1 promoter in long-term application, since it is not silenced [41].

**Figure 4:**
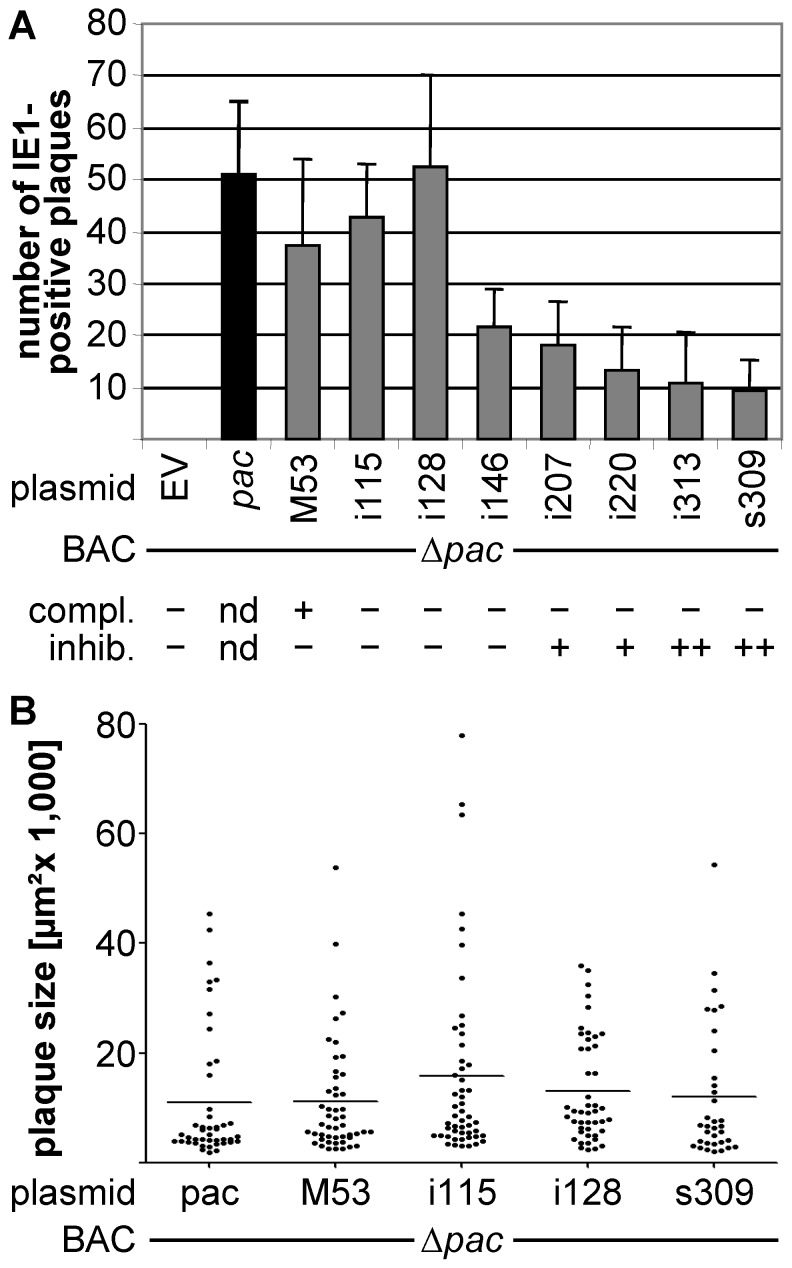
Complementation of the *pac* deletion and validation of the cell-based inhibitory screen. **(A)** Flpe-NIH were nucleofected with the Δ*pac* BAC and either an empty control shuttle vector (EV), a rescue plasmid carrying the *pac* sequences (*pac*, black bar), or a rescue plasmid carrying the *pac* plus different M53 mutants, mixed with NIH/3T3 and fixed at 6 dpt. Cells were fixed with an IE1-specific antibody and the number of IE1-positive plaques was quantified. Depicted are mean and SD of triplicate values of three independent experiments. M53 mutants were derived from a transposon-based random mutagenesis described and characterized in [17], [33]. Their capacity to complement the wt pM53 function of a ΔM53 virus (compl.) and to inhibit the wt pM53 function in a dominant negative manner (inhib.) is indicated below the diagram. **(B)** Flpe-NIH were treated as described in (A). Images of IE1-positive foci were taken and the size of the plaques determined using the area measuring tool of the imageJ software (http://imagej.nih.gov/ij/). Depicted are values and mean of plaques derived from two experiments. The numbers of the analyzed plaques for pac, n = 44; M53, n = 49; i115, n = 51; i128, n = 46; s309, n = 34.

First, we investigated whether the deletion of the predicted packaging signal will indeed prevent virus reconstitution from the mutant BAC Δ*pac*. To test this, MEF were transfected with two independent Δ*pac* BAC clones and the production of viral progeny monitored. In both cases no viral plaques were observed for six weeks, confirming that the *pac* sequences were crucial for virus formation (data not shown). Next, we tested whether virus reconstitution was possible from *pac*-lacking genomes by cell-based re-insertion of the packaging signals from pDEST-pac. For this, Flpe-NIH cells were nucleofected with the Δ*pac* BAC and either an empty vector or the *pac*-harboring rescue plasmid and viral plaques quantified at 6 dpt. No viral plaques were detected after nucleofection with the Δ*pac* BAC and the empty vector. In contrast, complementation using the *pac*-containing pDEST-pac rescue plasmid resulted in robust plaque formation, ranging between 40 and 60 plaques per sample (Fig. 4A).

Since it was shown that the *pac* signals were crucial for viral reconstitution and that their deletion from the MCMV genome could be complemented using the CCA, the usefulness of the approach for the identification of inhibitory mutants was tested. Again, the M53 mutant library was used as reference set. A number of mutants unable to complement the M53 deletion BAC [33], but with diverse potential to inhibit the wt pM53 function upon their overexpression [17] were chosen and tested using the *pac-*based CIA (Fig. 4A).

Between 30 and 60 IE1-positive plaques were observed upon expression of wt pM53 from the *pac*-based rescue plasmid in addition to the endogenous pM53. The mutants i115, i128 and i146 were non-functional, but did not interfere with wt pM53 in the previous screen. In accordance to these expectations, plaque numbers comparable to the wt control were detected after co-nucleofection of rescue plasmids expressing either i115 or i128 mutants. Interestingly, only about half as much plaques formed in the presence of the i146 expressing rescue plasmid. The mutants i207 and i220 were known to be partially inhibitory, i.e. reconstitution was delayed using the previous assay [17]. In the presence of either mutant less than half of the plaque numbers of the wt M53 control were observed. Unexpectedly, around 10 plaques could be detected in the presence of the i313 and s309 mutants, respectively. Both proteins were shown to have a strong inhibitory potential and, when conditionally expressed in the wt genome context, could inhibit MCMV replication up to a millionfold [17].

Furthermore, we determined the sizes of the produced plaques. For this, Flpe-NIH cells were nucleofected with representative samples of the CIA validation including complementing (i115), non-complementing/non-inhibitory (i128) and non-complementing/inhibitory (s309) mutants of M53 and the necessary controls (wt M53 expression vector and empty vector) according to the basic protocol Here, however, we used an FCS for the overlay media which was selected for slow growth of mouse fibroblasts.At 6 dpt we fixed the cultures and microscopic images of IE1-positive foci were recorded. The rim of the plaques was outlined and the surrounded area determined applying the measuring tool of the imageJ software [42]. As it is shown in Fig. 4B no significant differences could be observed between wt and mutant versions of M53…

Taken together, using the M53 mutant library as reference set, the CIA could identify inhibitory mutants. However, in contrast to the CCA, where non-functional mutants could not induce plaque formation, here the previously known inhibitory mutants merely reduced the plaque formation efficiency to less than half of the value of the wt M53 control. Thus, using a cut-off value of 50% of the control, it would be possible to identify inhibitory mutants.

### Application of the CCA to study pM99

To validate the cell-based complementation approach further, we chose pM99 for mutagenesis. The M99 ORF is small and information about functionally important motifs were published for its HCMV homologue pUL99 [43]–[45], making this gene attractive for a test run. At first, we tested whether pM99 was an essential gene, as it was described for its HCMV homologue pUL99 by Britt and colleagues [46], or whether it was not essential for virus spread as reported by Silva *et al.* [47]. To this end, an M99 deletion BAC (ΔM99) was generated. To test the replicative capacity of MCMV in the absence of pM99, MEF were transfected with the ΔM99 BAC and plaque formation monitored. Whereas viruses were reconstituted from the wt MCMV BAC within a few days, no viral progeny was detected originating from the ΔM99 BAC during six weeks, indicating that pM99 was critical for virus production. To confirm that the observed reconstitution defect was in fact due to the loss of pM99, a pM99 expression plasmid was inserted ectopically into the ΔM99 BAC by Flp/FRT-mediated recombination in *E.coli* and used to transfect MEF. In this case, viral plaques were detected within 5 dpt and complete cell lysis was reached within two weeks (data not shown).

**Figure 5:**
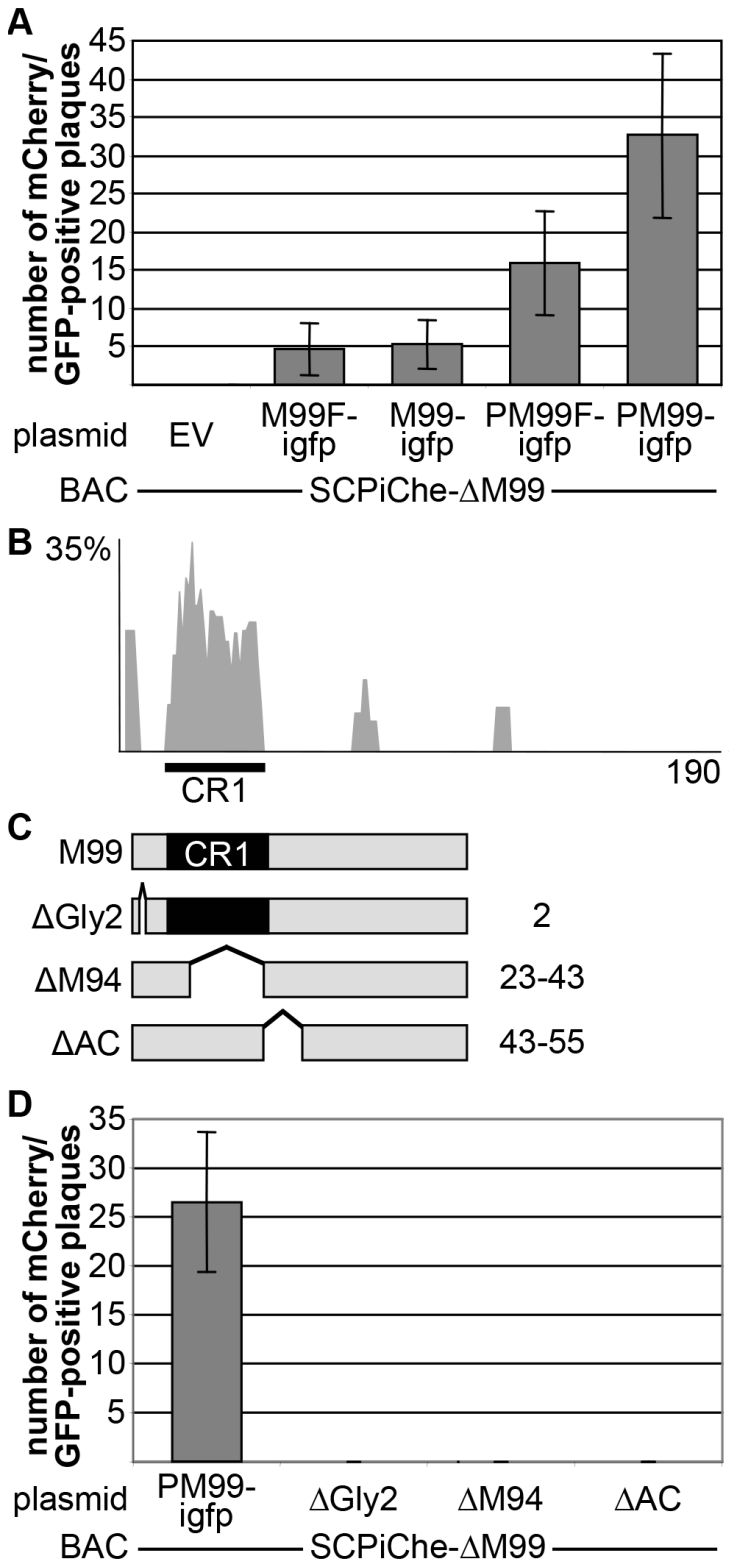
Construction and CCA complementation of pM99 mutants. **(A)** Flpe-NIH were nucleofected with the indicated BAC and plasmids and plaque formation quantified at 6 dpt. F, Flag-tag; P, P_M99_. Depicted are mean and SD of duplicate samples of two independent experiments. **(B)** The amino acid sequences of 34 pUL11 homologues (accession numbers are listed in Table S2) were aligned using the Vector NTI AlignX program (Invitrogen) via the BLOSUM 62 similarity matrix. The similarity plot was calculated using a 5 amino acid window size, with scores for weak and strong similarity and identity of 0.2, 0.5, and 1.0, respectively. The *x* axis represents the number of amino acids in the consensus sequence. The conserved region (CR) is indicated below the diagram. **(C)** Schematic overview of the pM99 mutants. Proteins are depicted as gray bars, the conserved region (CR1) are indicated as black boxes. Deletion mutants are shown with bridged spacing. Numbers on the right indicate the amino acid of pM99 affected by the mutation. **(D)** Flpe-NIH cells were treated as in (A). Mutants are based on the PM99-igfp plasmid. Depicted are mean and SD of duplicate samples of two independent experiments.

Since pM99 appeared to be essential for MCMV and ectopic insertion by the traditional Flp/FRT technique restored viral growth, we tested the M99 complementation using the CCA. To facilitate automated quantification using fluorescent signals, a ΔM99 acceptor BAC was used, in which mCherry expression was coupled by an internal ribosomal entry site (IRES) to the endogenous expression of the smallest capsid protein (SCPiChe-ΔM99). In the rescue plasmid we fused a GFP ORF to the M99 expression cassette via an IRES to monitor the successful integration of the complementing plasmid by fluorescent microscopy. First, we tested the M99 complementation using different control elements for the M99 expression. We constructed rescue plasmids expressing pM99 under control of the CMV promoter (P_CMV_) or under control of the endogenous M99 promoter (P_M99_). We also wanted to test how Flag-tagging of pM99 would influence virus reconstitution. To this end, we generated C-terminally tagged variants and inserted them into the rescue plasmids with both promoters. Flpe-NIH were nucleofected with the SCPiChe-ΔM99 BAC together with each of the pM99-expressing plasmids and viral dual-color plaque formation was quantified at 6 dpt (Fig. 5A).

Whereas no viral progeny was observed upon nucleofection of SCPiChe-ΔM99 together with an empty rescue vector, confirming that lack of M99 does not allow virus reconstitution, co-transfection with each of the pM99-expressing rescue vectors resulted in plaque formation. In comparison, complementation with pM99 expressed under control of PCMV was less efficient and Flag-tagging had also a negative effect on reconstitution frequency. As two extremes, only few plaques were detected upon PCMV-regulated pM99F expression, indicating that excess pM99 and pM99F obstructed virus production. In contrast, PM99-driven expression of pM99 restored viral reconstitution more efficiently, yielding much higher plaque numbers (Fig. 5A).

Taken together, this analysis successfully established the complementation of the M99 deletion, providing insights into the construction and investigation of potential pM99 mutants, namely to preferentially use constructs expressed under control of the M99 promoter.

Since a comprehensive mutant library covering the M99 ORF is not yet available, a couple of mutants were generated based on the information available for the HCMV pUL99 protein. Alignment of 35 representative pUL11 homologues revealed just one homology peak, designated CR1, although the maximum similarity was quite low (Fig. 5B). In HCMV pUL99, this region contains the pUL94 binding motif [45]. Furthermore, the N-terminal glycine was shown to be myristoylated and amino acids 44–57 comprise an acidic cluster (AC). Both features are required for correct localization of the protein and viral growth, whereas the C-terminal part is dispensable [43]. Corresponding to this, pM99 mutants were generated lacking the first glycine (ΔGly2), the potential pM94 binding domain (ΔM94) or the acidic cluster (ΔAC) (Fig. 5C). The expression of all mutants was regulated by PM99.

Nucleofection of Flpe-NIH cells with the SCPiChe-ΔM99 BAC together with one of the mutant M99-expressing plasmids repeatedly did not result in the formation of viral progeny, whereas reconstitution was observed in the control reaction using the wt M99-expressing vector (Fig. 5D). This indicated that also in MCMV pM99 the three deleted features are crucial for the protein functionality.

## Discussion

Identification of DN proteins can be separated into consecutive steps. At first, a mutant library of an essential gene of interest is generated. Traditionally, this is carried out using random transposon mutagenesis, yielding numerous candidate genes. These are inserted into a viral BAC deficient for this gene, and analyzed with respect to their ability to restore virus reconstitution. Mutants that fail to complement the wt gene point out key regions of the protein. The DN potential of those mutants is then tested by their expression in the wt genome context [16]. The time expenses of this scheme can be reduced in two aspects. First of all, the number of mutants to begin with can be reduced by disrupting different regions of the protein by targeted mutagenesis as described for pM53 [15]. Thereby, essential domains are identified by investigating only a limited number of mutants, which can be subjected on demand to a more thorough mutagenesis with an even higher coverage of the parts of interest. Where a targeted mutagenesis is not possible, the alternative approach would be to test many mutants in a high-throughput screening (HTS) system. To this end, we developed a host cell-based flip-in system that omits the necessity to manipulate the MCMV genome in bacteria.

In the classical approach, the expression cassette encoding the (mutant) gene of interest was inserted into the MCMV BAC via site-specific recombination, which was performed in bacteria and demanded the subsequent purification of recombinant BACs from a bacterial culture. In the new system, the cell-based complementation assay (CCA), we replaced this step by allowing recombination to take place within the mammalian target cells, which were modified to stably express Flpe recombinase, an evolved form of the original Flp recombinase better adapted to catalyze the recombination reaction at 37°C [30]. The presence of the Flpe-encoding mRNA, i.e. the indicator for active transcription, was confirmed by reverse transcription of isolated RNA and PCR in parallel to each experiment and was detectable for several weeks of sub-culturing. Additionally, Flpe functionality was confirmed using a plasmid-based recombination assay. Although substantial recombination was observed, not all transfected plasmids were converted. This might in part reflect the equilibrium of flip-in and flip-out, as reported for this recombinase [48]. However, since we did not analyze recombination on the single cell level, it is also possible that some cells in the Flpe-NIH population fail to express functional Flpe. Nonetheless, if that was the case, the portion of functional Flpe-NIH was sufficient for our assays.

The Flpe-NIH were transfected using a technology termed nucleofection, which facilitates the direct ingress of the transfected DNA into the nucleus of the treated cell [36], [37], [38]. This provides the chance to transfect even non-dividing cells, which is not an issue for the NIH/3T3 cell line, from which the Flpe-NIH are derived, but which is quite useful for MEF, which do not divide any more after reaching complete confluence. Another advantage of this technique is that it reduces the effort to transfect high numbers of samples under comparable conditions. In comparison, for transfection reactions utilizing lipids or liposomes the DNA has to be mixed with the transfection reagent and added onto the cells or into the cell culture medium. This requires the successive preparation of one reaction mix per sample. For nucleofection, cells are harvested and re-suspended within the transfection solution, before the whole mixture is added to the DNA preparation. That not only grants analogous treatment and conditions for the cells, which are handled as one block, but also permits to set up up to 96 samples in parallel in a much shorter time frame.

The CCA was successfully tested and evaluated using BACs devoid of an essential viral gene or genetic feature as well as already characterized mutants of an M53 library created by random mutagenesis [33]. Whereas nucleofection with the ΔM53, the ΔM56 and the ΔM104 BAC together with an empty rescue vector did not produce viral progeny, the complementation with the respective wt genes reproducibly resulted in plaque formation. The same was true for the complementation of the deletion of the *pac* sequences, which are essential for encapsidation and cleavage of newly replicated MCMV genomes into the viral capsids [40]. Nucleofection with the Δ*pac* BAC and the respective *pac*-containing rescue plasmid successfully restored virus production, resulting in 40 to 60 plaques per sample. This confirmed that the CCA is feasible for virus reconstitution. Furthermore, the results are quantifiable already at 6 dpt, the transfected cells do not need to be maintained for 6 weeks. Possibly, mutations that are partially functional but lead to delayed virus reconstitution may be negative in this screen. These, however, can be analyzed in more detail in the inhibitory screen. Altogether, the cell-based complementation assay is performed much faster than the traditional complementation using the bacterial flip-in, reducing the required time by several weeks.

It should be noted that the virus we used in our study is a mutated form of MCMV lacking the genes *m01* to *m16*. These are dispensable for viral growth in cell culture and the resulting virus has wt-like properties *in vitro* [19]. Although unlikely, it cannot be absolutely excluded that the combined loss of those genes together with an essential gene may have an unanticipated effect. This, however, cannot be assessed in full detail since any mutant MCMV lacking an essential gene will not produce any virus, irrespective of the presence or absence of the first 16 genes. We believe that a robust basis to test mutants is given if virus growth can be restored by ectopic insertion of an expression cassette of the deleted gene. Negative or cumulative effects of parallel deletions will not be identifiable using our assay or simple recombination tests. It might be that mutants are less complementary (in the CCA) or more inhibitory (in the CIA) than they would be using the wt background. If this is suspected, potential DN candidates could be verified comparing the effect of conditional mutant expression in the wt and the deletion background. So far, we did not observe any such effects (unpublished observations).

Since the CCA system was not only developed to reconstitute viruses, but to identify non-complementing mutations in a target gene, we tested it using a reference set of M53 mutants [33]. According to the expectations, the complementing mutants (i43, i104) led to the formation of viral progeny, whereas the non-functional mutants (i128, i212) as well as the inhibitory mutants (i207, s309) did not induce virus production. These observations confirmed the applicability of the CCA to identify non-complementing mutations that might be inhibitory for viral growth.

In the next step, we applied the new system to isolate inhibitory mutations. For this, a set of M53 mutants that have been shown to be non-complementing for the M53 deletion BAC [33], but with diverse potential to inhibit the wt pM53 function upon their overexpression [17] were tested using the *pac-*based inhibitory assay (CIA). In agreement with the expectations, expression of non-functional but non-inhibitory mutants (i115, i128, i146) did not interfere with viral growth and resulted in plaque numbers comparable to wt pM53 overexpression, whereas expression of the partially inhibitory mutants (i207, i220) decreased viral plaque formation to less than half of the control. However, unexpectedly the known DN proteins (s309, i313) were not able to inhibit virus production completely, as it has been shown previously using constructs which were fully constructed in *E. coli*. [17]. In the assay presented here the decisive genetic recombination is performed in the host cells in which the virus reconstitution takes place. It is possible that escape mutants are generated which do not carry or express the inhibitory mutants but maintain the cis element from the rescue plasmid which is required for plaque formation. It is also possible that in our assay the inhibitory potential of the DN alleles is weaker than in the original assays. This is however unlikely because only reduction of the plaque numbers but not a reduction in average plaque size was observed for the inhibitory mutant, indicating that the frequency of the reconstitution was affected and the inhibitory feature was not maintained after infectious virus was reconstituted.

The inhibitory potential could also not be increased by exchanging the expression-regulating promoter. This might require closer investigation with regard to the expression levels of the mutant gene products, for example by quantitative PCR or Western blot analysis. Nevertheless, the CIA successfully made use of an essential genetic element that cannot be complemented in *trans* (the *pac* sequences), thereby supporting the integration of the rescue plasmid. Using the M53 mutant library as reference set, the results were not as clear cut as we expected, as the known strong DN examples i313 and s309 did not completely prevent plaque formation. However, the plaque numbers quantified for all the inhibitory mutants were considerably less than half of the value of the wt pM53 control. Thus, using a cut-off value of 50% of the control it was possible to identify inhibitory mutants. Clearly, the DN potential of all identified inhibitory mutants needs to be analyzed by further assays.

Based on previously characterized M53 mutants, it was demonstrated that the CCA was applicable to identify non-complementing mutation within a target gene. However, to show its reliability, we wanted to test the system by screening mutants of a hitherto not analyzed gene. For this, we chose the MCMV M99 ORF, which encodes a small protein of 112 amino acids. pM99 belongs to the conserved family of pUL11 homologues, which were, together with the homologues of pUL16 (pM94 in MCMV), implicated in the process of secondary envelopment of tegumented viral capsids within the cytoplasm [19], [22], [24], [45], [49].

Deletion of the M99 ORF from the wt MCMV BAC hindered virus reconstitution in transfected MEF, a defect that was reversed by pM99 expression at an ectopic position in the genome. This indicated that pM99 is essential for viral growth, which has also been observed for the HCMV homologue pUL99 [50], [51]. This is in contrast to alpha-herpesviruses as well as EBV, where inactivation of the homologous ORFs did not prevent virus formation, although plaque sizes and viral titers were reduced [24], [49], [52], [53], suggesting that the functions required for secondary envelopment are carried out at least in part redundantly in those viruses. Subsequently, the M99 deletion was *cis*-complemented using the CCA system. The deletion of pUL99 in HCMV did not prevent virus spread after infection with the *trans*-complemented deletion mutant virus [47]. Here, in our *cis*-complementation assay for MCMV, we did not observe virus spread in the absence of functional pM99. This is in accordance with the results published by the deletion screens for HCMV, which also involved virus reconstitution in their assays [50], [51].

Since it was not known, whether pM99 overexpression would be detrimental for the reconstituting virus, expression cassettes using the endogenous M99 promoter (P_M99_) and the strong CMV immediate early promoter (P_CMV_) were tested. Although complementation with either construct resulted in viral plaque formation, pM99 expression under control of the P_M99_ was much more efficient in facilitating viral growth. Reconstitution of viruses from isolated DNA may be affected at several stages, possibly due to deregulation of viral gene expression upon overload with viral genomes or due to the toxicity caused by overexpressed viral proteins. Abundant expression of certain viral proteins, particularly early expression of proteins with late kinetics, may interfere with virus replication. The data obtained for the pM99 complementation strongly highlight the necessity of tightly regulated protein expression, as is achieved in wt MCMV [54].

Supportive observations were made for the bacterial flip-in of a P_CMV_-driven pM99 expression cassette into the ΔM99 BAC. The recombinant BAC failed repeatedly to reconstitute virus (data not shown). Thus, a major conclusion that can be drawn from the CCA is that early and unregulated expression of pM99 is inhibitory for virus production. In contrast to that, pM53 and pM94, which are also expressed with late kinetics [19], [33], do not impair viral replication when expressed early and in excess [15], [19].

In addition to the basic cis-complementation with the wt gene, we constructed three mutants based on functional domains described for the HCMV homologue pUL99 to validate the novel system. These included the potential myristoylation site, a conserved acidic cluster and the potential pM94 binding domain [43], [45], which were deleted from the M99 ORF. Repeatedly, none of these mutants was able to complement the wt pM99 function, indicating that those domains are as well important for the functionality of pM99. In the next step, the three mutants should be tested in the inhibitory screen to check whether the mutations result in nonfunctional proteins, which cannot interfere with wt pM99, or whether the mutant proteins are inhibitory for MCMV replication.

Additionally, complementation of the M99-deficient BAC demonstrated that the rescue plasmid harboring the *pac* sequences can be utilized in the CCA as well. Reconstitution of the ΔM99 BAC with an ectopic flip-in of this plasmid (ΔM99EPM99) produced viral progeny that could productively infect a new cell generation, indicating that a second *pac* signal does not result in the formation of defective MCMV genomes. Due to this observation, it is possible to use the same rescue plasmid as acceptor for the mutant genes in both screens, the complementary and the inhibitory one, which circumvents the re-cloning of non-complementing mutants of the first screen into a new rescue plasmid for the second screen.

Altogether, we report here on a novel complementation system that is suitable for the high-throughput screening to rapidly identify non-complementing mutations of essential MCMV alleles, which can then be tested in a second round for their inhibitory potential. Nevertheless, the inhibitory screen demands further improvement with respect to efficient flip-in and genome stabilization.

## Supporting Information

Table S1 **Oligonucleotides.**
(DOC)

Table S2 **Accession numbers of pUL11 homologue sequences.** Listed are the accession numbers of the pUL11 homologues used for the alignment depicted in Figure 5B. Protein sequences were downloaded from the Protein Knowledgebase (UniProtKB) on http://www.uniprot.org.
(DOC)
