# Supplementary material for: A Modified Screening System for Loss-of-Function and Dominant Negative Alleles of Essential MCMV Genes
Source: PLoS One. 2014 Apr 14;9(4):e94918. doi: 10.1371/journal.pone.0094918 (PMC3986410; doi:10.1371/journal.pone.0094918)
Supplement: Table S1 — Oligonucleotides. (DOC) [file pone.0094918.s001.doc]

**Table S1: Oligonucleotides**

| **Name** | **Sequence (5’-3’)** |
| --- | --- |
| BLAfor | GCCGGGAAGCTAGAGTAAGTAGTT |
| BLArev | GGTCGCCGCATACACTATTCTC |
| Flpe-for | ATGGCTCCCAAGAAGAAGAG |
| Flpe-rev | GTGATCTCCCAGATGCTTTC |
| MCMVpac_for | GTGTGTCGGACCGGGTTTAGATAAAAATGTGACCG |
| MCMVpac_rev | GTGTGTCATATGAAAACCCTGGACTCCCCCGAAC |
| M99ΔAC-5’rev | AGACGTATCCGTCAGCACAGAGAATTCGG |
| M99ΔAC-3’for | TTCGTGCAGAAACAGCTCACGAC |
| M99ΔGly2-for | GCCGCTAGCGGTACCATGGCAGAGTGCTGTAAACAGC |
| M99ΔM94-5’rev | CAGGGAGTCCGCGGCGTAGGG |
| M99ΔM94-3’for | GACGAAGACCAGGTCGGCG |
| M99rev | TTTAGGCCTGTCGACTCACAAGGCCCTGACTTTTTTCTTC |
| M99syn-for | AAGGCGCGCCGCTAGCGGTACCATGGGTGCAGAGTG |
| NR15rev | AATGACCGACCAAGCGACGCCC |
| P(M99)-for | GCTTAATTAACTGTTCCTCAAGGCCGCGTTC |
| P(M99)-rev | TTTGCTAGCAGCGCGAGACCCGTCTTCTTAC |
| REC15for | CGGGCCTCTTCGCTATTACGCC |
| REC15rev | TGGCACGACAGGTTTCCCGACT |
| H5-DPAC | CTCGTTAACCGAGCACATGTTTTTTTAACGACTCCTCCACACACATATGATGTGGGCGGACAAAATAGTTGG |
| H3-DPAC | GGGTACCGAGCTCGAATTCACTGGCCGTCGTTTTACAACGTCGTGACTGGGTGTGGGCGGACAATAAAGTCTTAAACTGAA |
| H5-M56 | CGTCTCGTTATATAGTGCGACGGACAGGACGAGCTCTCGAATGTGCGCCCTGTGGGCGGACAATAAAGTCTTAAACTGAA |
| H3-M56 | AAGCGGAGGGGTAGGGGAGTCGACCTGCTCGCGCGCAACGTCGGCAAGGTGTGGGCGGACAAAATAGTTGG |
| H5-M99amp | GTGGGTGCAGAGTGCTGTAAACAGCTATGTCGCAGCCTGCATCCCTACGCCGCGGACTCCCTGAGAACCATCACCCTAATCAAGTTT |
| H3-M99amp | CTCTTTCTCCCTTTCTCCCCCCCTCACGGTCGATCGATAGATAGATATCAGAGTAAACTTGGTCTGACAGTTACC |
