# Supplementary material for: A Modified Screening System for Loss-of-Function and Dominant Negative Alleles of Essential MCMV Genes
Source: PLoS One. 2014 Apr 14;9(4):e94918. doi: 10.1371/journal.pone.0094918 (PMC3986410; doi:10.1371/journal.pone.0094918)
Supplement: Table S2 — Accession numbers of pUL11 homologue sequences. Listed are the accession numbers of the pUL11 homologues used for the alignment depicted in Figure 5B. Protein sequences were downloaded from the Protein Knowledgebase (UniProtKB) on http://www.uniprot.org. (DOC) [file pone.0094918.s002.doc]

**Table S2: Accession numbers of pUL11 homologue sequences**

Listed are the accession numbers of the pUL11 homologues used for the alignment depicted in Figure 5B. Protein sequences were downloaded from the Protein Knowledgebase (UniProtKB) on http://www.uniprot.org.

| A5A411_9ALPH | Q5Y0U2_9ALPH | Q9QP14_ILTV | TG11_PSHV1 |
| --- | --- | --- | --- |
| D1FXX1_FHV1 | Q6X222_9ALPH | Q9YZA4_9ALPH | TG11_SHV21 |
| E2IUF9_SHV1 | Q77CB3_BHV1C | TG11_EHV1B | TG11_VZVO |
| F5HHY1_HHV8P | Q782T5_9ALPH | TG11_GAHVM | UL11_EBVA8 |
| G8H0M3_9BETA | Q806B8_CHV1 | TG11_HHV11 | UL11P_HCMVA |
| G8XT03_9BETA | Q85040_SUHVK | TG11_HHV2H | VG38_ALHV1 |
| G8XUG4_9BETA | Q8QS01_9BETA | TG11_HHV6U | VG38_ICHVA |
| G9CUB8_9ALPH | Q8UZG1_9GAMA | TG11_HHV7J |  |
| Q2QBG6_CHV16 | Q9E1H9_MEHV1 | TG11_MUHVK |  |
